# Anti-phospholipase A2 receptor antibody levels at diagnosis predicts outcome of TAC-based treatment for idiopathic membranous nephropathy patients

**DOI:** 10.1186/s12882-022-02914-4

**Published:** 2022-09-07

**Authors:** Bihua Wang, Zhidan Zhu, Feng Huang, Haowen Huang, Luxia Tu, Ying Wang, Linfeng Zheng, Jing Zhou, Xin Wei

**Affiliations:** 1 Department of Nephrology, The First Affiliated Hospital of Nanchang University, 17 Yongwai Zhengjie, Nanchang City, 330006 Jiangxi China; 2 Nanchang University, Nanchang City, 330006 China; 3 Department of Nephrology, People’s Hospital of Ganjiang New District, Nanchang City, 330006 Jiangxi China; 4 Pathology Department, The First Affiliated Hospital of Nanchang University, Nanchang City, China

**Keywords:** Anti-PLA2R antibody, Idiopathic membranous nephropathy, TAC, Treatment response, Outcome

## Abstract

**Background:**

Idiopathic membranous nephropathy (iMN) is recognized as an organ-specific autoimmune disease, mainly caused by anti-PLA2R antibody. This study aimed to study between anti-PLA2R antibody level at diagnosis and the response to tacrolimus (TAC)-based treatment in iMN patients.

**Methods:**

This was a retrospective cohort study including 94 kidney biopsy-proven MN patients with positive anti-PLA2R antibody at diagnosis from May 2017 to September 2021 in our center. All iMN patients received the TAC regimen as the initial immunosuppressive therapy. All patients were divided into two groups according to anti-PLA2R antibody titer at diagnosis: high-level group (> 150 RU/ml; *n* = 42) and low-level group (≤ 150 RU/ml; n = 52). The association between anti-PLA2R antibody levels and clinical outcomes was assessed using the Kaplan–Meier method.

**Results:**

The low density lipoprotein in the high-level group was significantly higher than low-level group at diagnosis, otherwise, serum albumin was significantly lower than low-level group; however, there was no significant difference in creatinine levels between two groups. The remission rates were significantly higher in the low-level group than high-level group after treatment with TAC for 12, 18, or 24 months (all *P* < 0.05). After 12 months of treatment with TAC, 82.7% of the patients in the low-level group achieved complete remission (CR) or partial remission (PR) (mean, 6.52 ± 0.53 months). However, 38.1% of the patients in high-level group achieved CR or PR (mean, 9.86 ± 0.51 months). Moreover, CR rate at 12 months in the high-level group was only 4.7% (mean, 11.88 ± 0.63 months). The infection frequency in the high-level group (35.6%) was higher than the low-level group (20%) during the TAC treatment, although there was no significant difference (*P* = 0.065). There were 19% patients who had end-stage kidney disease (ESKD), and 7.1% of patients died of ESKD in the high-level group during the follow-up period.

**Conclusion:**

Anti-PLA2R antibody level above 150 RU/ml at diagnosis can predict a poor treatment response and outcome of TAC treatment in iMN patients, who may not benefit from TAC or other calcineurin inhibitor regimens as the initial treatment.

## Introduction

Idiopathic membranous nephropathy (iMN) is a common cause of nephrotic syndrome in adults and accounts for 20% of primary nephrotic syndrome in China [1]. Nearly one-third of iMN patients can achieve spontaneous remission; in contrast, one-third of iMN patients with nephrotic syndrome level proteinuria (proteinuria > 3.5 g per 24 h and/or hypoalbuminemia) will progress to ESRD [2–4]. iMN patients with sufficient symptoms of nephrotic syndrome, such as edema, thrombotic events, and progression of kidney failure, and/or high risk of progression and/or low likelihood of spontaneous remission will require immunosuppressive therapy [5], which includes the use of alkylating agents, rituximab, CNI, or a combination of these agents.

Despite the higher complete or partial remission rates and lower relapse rates obtained with the use of alkylating agents (cyclophosphamide or chlorambucil) combined with steroids, its severe side effects such as infection, pancytopenia, and malignancies will prompt most physicians and patients to use rituximab or CNI as the initial treatment [6]. TAC is a type of CNI that is widely used to treat iMN patients alone or in combination with low doses of steroids [7]. Most studies have shown that TAC is effective and safe for the treatment of iMN [8, 9]. Despite the potential nephrotoxicity and high relapse rate after drug discontinuation, it was still recommended by the 2012 and 2021 Kidney Disease Improving Global Outcomes (KDIGO) guidelines as the first-line treatment for iMN patients [10, 11]. However, which iMN patients will benefit most from the TAC-based treatment or what will help inform the prognosis of iMN patients treated with TAC is still unclear.

The discovery of the anti-PLA2R in 2009 provided evidence that iMN is an organ-specific autoimmune disease [12]. Anti-PLA2R antibody is present in 70%–80% of patients with iMN, and has more than 95% specificity [13–16]. Researches indicate that the titer of anti-PLA2R antibody is highly associated with disease severity and prognosis, which mean that a high level of anti-PLA2R antibody always has a worse therapeutic response [17–20], and lower spontaneous remission [17, 21–23], meanwhile, the depletion of anti-PLA2R antibody is usually followed by a clinical remission of nephrotic syndrome. However, the role of anti-PLA2R antibody titer in the individualization of immunosuppressive therapy remains unclear. According to the draft version of the 2020 KDIGO clinical practice guidelines, iMN patients with anti-PLA2R antibody titers > 150 RU/ml are considered to be at high risk of disease progression, and should consider the need to start immunosuppressive therapy including rituximab or glucocorticoids with cyclophosphamide or CNI-based therapy. However, the therapeutic response to TAC, which is the most widely used CNI regimen in high-risk patients with iMN, has not been well demonstrated.

Thus, we conducted this retrospective study to compare the effect and tolerance of TAC-based therapy in iMN patients with high anti-PLA2R titer (> 150RU/ml) and low anti-PLA2R titer (≤ 150RU/ml).

## Method

### Patients

**Fig. 1:**
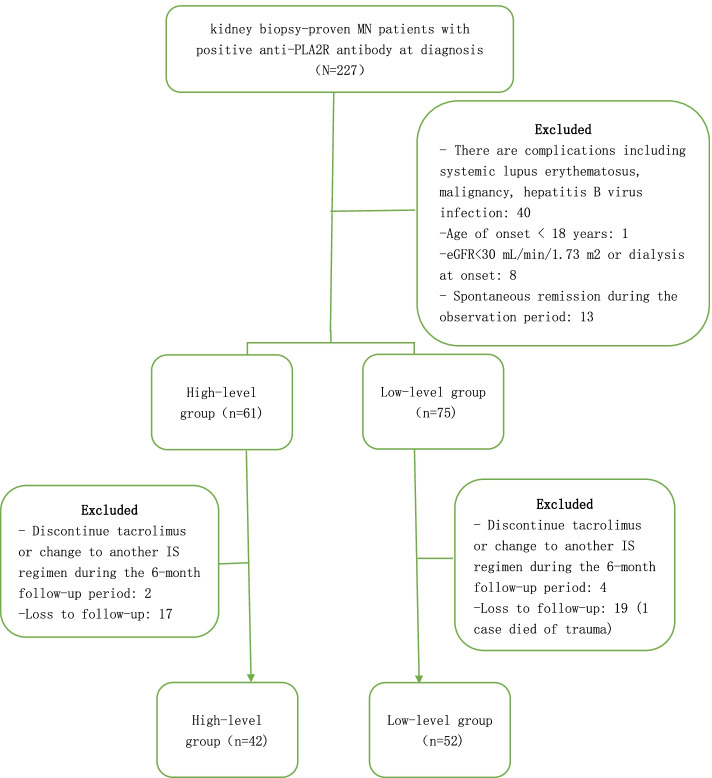
Inclusion flowchart of patients with idiopathic membranous nephropathy

All patients were diagnosed, treated and followed up at the First Affiliated Hospital of Nanchang University (Nanchang, Jiangxi Province, China) from May 2017 to September 2021. In this retrospective study, we collected 227 adult patients with membranous nephropathy (MN) by clinical diagnosis. The inclusion flowchart of patients with iMN is presented in Fig. 1. The inclusion criteria were listed in the flow chart (Fig. 1). All included cases were recorded in the Human Genetic Hospital of Nanchang University. All iMN patients were divided into two groups according to anti-PLA2R antibody titer at diagnosis: high-level group (> 150 RU/ml; *n* = 42) and low-level group (≤ 150 RU/ml; *n* = 52).

### Date collection

Anti-PLA2R antibody titers were detected by enzyme-linked immunosorbent assay (ELISA) kits *(EUROIMMUN)* in our center. According to the manufacturer’s instructions, a positive antibody titer was defined as an anti-PLA2R antibody titer of > 20 RU/ml. The limit of quantitation was equal to 2.0 RU/ml. ELISA was performed according to the manufacturer’s instructions. Human serum samples diluted to 1:101 in sample buffer were added to microtiter wells and then incubated for 30 min at room temperature (+ 18 to + 25 °C). The antibody was detected by incubating the samples with enzyme-labeled anti-human IgG for 30 min, after which chromogen substrate solution (EUROIMMUN AG) was added to each well. Optical density was measured using an automated spectrophotometer (1510; Thermo Fisher Scientific, Waltham, MA, USA) at 450 nm.

Data on patient characteristics, treatment responses, complications and outcomes were obtained by reviewing the patients’ medical records. The clinical data of all patients collected were as follows: age at diagnosis, sex, weight, blood pressure, specific diagnosis, follow-up symptomatic and immunosuppressive therapy (drug, dose, time and duration). The laboratory data were continuously followed to September 30, 2021 or the occurrence of endpoints, including serum albumin (Alb), serum creatinine (Scr), 24-h urine protein (24hUP) quantity, ratio of urine protein to urine creatinine, total cholesterol (TC), triglyceride (TG), hemoglobin (Hb), fasting blood glucose (FBG), and infection-related indicators. Estimated glomerular filtration rate (eGFR) was calculated using the Chronic Kidney Disease Epidemiology Collaboration (CKD-EPI) equation. The main complications and drug-related side effects include infection, thromboembolism, acute kidney injury (AKI), ESRD, diabetes/glucose intolerance. The main endpoints included death, loss of follow-up, and the last follow-up date. The last follow-up date of the patients in the study was September 30, 2021. The follow-up period of patients ranged from 6–42 months (mean, 19.76 ± 9.30 months).

### Evaluation criteria

The primary outcome included CR and PR at 6, 12, 18 and 24 months follow-up. The secondary outcomes included remission time, infection, secondary abnormal blood glucose, renal dysfunction, death and relapse. CR was defined as proteinuria < 0.3 g/24 h observed on at least three consecutive visits and a normal range of serum albumin and creatinine. PR was defined as proteinuria < 3.5 g/24 h and at least 50% reduction from the time of initiating conservative treatment, serum albumin > 30 g/L, and stable creatinine [11]. Total remission was defined as CR and PR. Relapse was defined as proteinuria > 3.5 g/L/24 h on at least two consecutive tests after CR or PR. AKI can be diagnosed clinically if it meets the following criteria: ① the increase of SCR ≥ 0.3 mg/dl within 48 h; ② Confirm or speculate that SCR increases by ≥ 50% compared with the basic value within 7 days ③ urine volume is less than 0.5 ml/kg/h, lasting for ≥ 6 h [24]. Oliguria is defined as urine volume less than 400 ml per 24 h in adults. Anuria is defined as urine volume less than 100 ml per 24 h in adults. ESKD is defined as an eGFR < 15 mL/min/1.73 m2 (CKD stage 5) or the necessity for dialysis/transplantation. Treatment failure was defined as the absence of CR or PR during the follow-up period.

### Statistical analysis

SPSS 21.0 statistical software was used for data analysis. Data were expressed as mean ± SD and compared by *T-test* if normally distributed. The measurement data are expressed as the median (lower quartile, upper quartile) and compared by Wilcoxon rank sum test if not normally distributed. The Categorical variables were expressed by frequency and percentage, and compared by Chi-squared’ test, Fisher’s exact test. Survival and cumulative remission curves were drawn using the Kaplan–Meier method.

## Results

Ninety-four kidney biopsy-proven primary MN patients with positive serum anti-PLA2R antibody titer at diagnosis were enrolled finally in the retrospective study, with more males than females, and a mean age of 57 years. The baseline characteristics of patients with iMN at baseline in the high-level group and low-level group are presented in Table 1. The antibody titers of the patients were 72.20 (39.59, 118.80) in low-level group and 374.31 (262.50, 592.63) in the high-level group. The mean age of patients in the low-level group was 55.27 ± 11.74 years, is not significantly different from the high-level group (59.38 ± 13.05 years, *P* = 0.112). The baseline 24- hour urinary protein of the low-level group was 4.47 ± 1.64 g, and that of the high-level group was 5.02 ± 2.86 g. There was no significant statistical difference in 24-h urinary protein levels between the two groups (*P* = 0.226). At diagnosis, the serum albumin in the high-level group was significantly lower (*P* = 0.005), and low-density lipoprotein was significantly higher than low-level group (*P* = 0.010). There was no significant difference in serum creatinine, and eGFR at diagnosis between the two groups.

**Table 1:** Baseline characteristics of iMN patients between low-level and high-level group

Parameters	Low-level group(*n* = 52)	High-level group(*n* = 42)	*P* value
Level of anti-PLA2R Ab, RU/mL	72.20(39.59, 118.80)	374.31(262.50, 592.63)	** < 0.001**
Gender, male/female	35/17	24/18	0.311
Age, year	55.27 ± 11.74	59.38 ± 13.05	0.112
Weight, kg	65.24 ± 9.06	63.52 ± 8.73	0.355
Systolic blood pressure, mmHg	131.27 ± 16.08	132.55 ± 19.71	0.730
Diastolic blood pressure, mmHg	83.08 ± 9.52	82.48 ± 8.41	0.750
Hemoglobin, g/L	128.07 ± 22.86	124.45 ± 14.61	0.355
Proteinuria, g/24 h	4.47 ± 1.64	5.02 ± 2.86	0.266
Total cholesterol, mmol/L	7.78 ± 2.86	8.71 ± 2.46	0.102
Triglyceride, mmol/L	2.39(1.65, 3.88)	2.28(1.58, 3.43)	0.533
Low density lipoprotein, mmol/L	4.70(3.47, 6.45)	6.18(4.53, 8.01)	**0.010**
Total protein, g/L	47.24 ± 7.53	45.39 ± 6.20	0.205
Serum albumin, g/L	23.43 ± 5.44	19.75 ± 4.31	**0.005**
Blood urea nitrogen, mmol/L	5.25(4.33, 6.38)	5.05(4.08, 6.35)	0.465
Serum creatinine, μmol/L	73.20(63.83, 86.08)	72.85(60.08, 91.48)	0.781
Trough blood concentration of TAC	5.82 ± 1.44	5.71 ± 1.51	0.733
eGFR, mL/min/1.73 m^2^	89.59 ± 17.41	85.75 ± 21.63	0.287
ACEI/ARB	48/52	40/42	0.878

Bold indicate *p* value < 0.05

Legend: *anti-PLA2R Ab* anti-phospholipase A2 receptor antibody, *iMN* idiopathic membranous nephropathy, *eGFR* estimated glomerular filtration rate, *low-level group* anti-PLA2R antibody titer ≤ 150 RU/ml, *high-level group* anti-PLA2R antibody titer > 150 RU/ml, *ACEI* angiotensin-converting enzyme inhibitor, *ARB* angiotensin receptor blocker

### Association between anti-PLA2R Ab levels and clinical remission

**Fig. 2:**
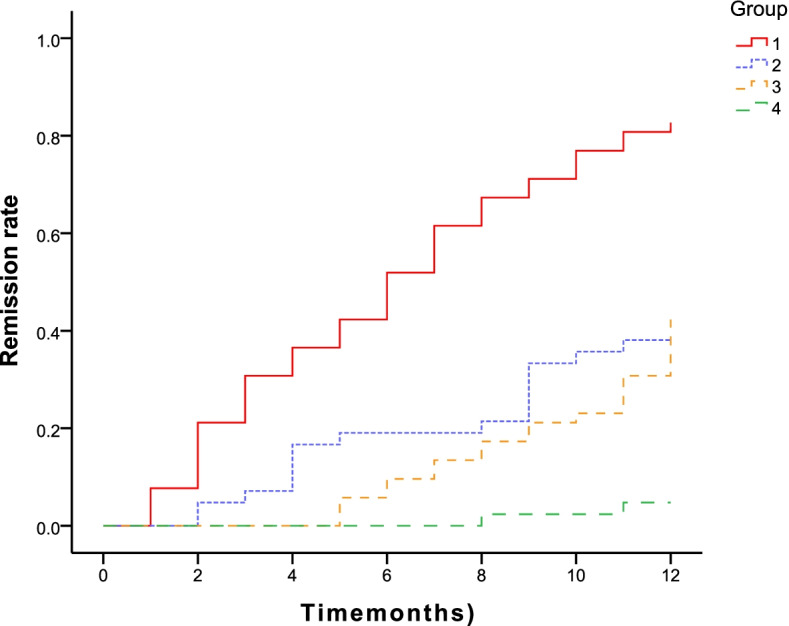
Remission rate is depicted for iMN patients in two groups at 1 year. The line 1 means total remission rate in low-level group, the line 2 means total remission rate in high-level group, the line 3 means complete remission rate in low-level group, the line 4 means complete remission rate in high-level group

**Fig. 3:**
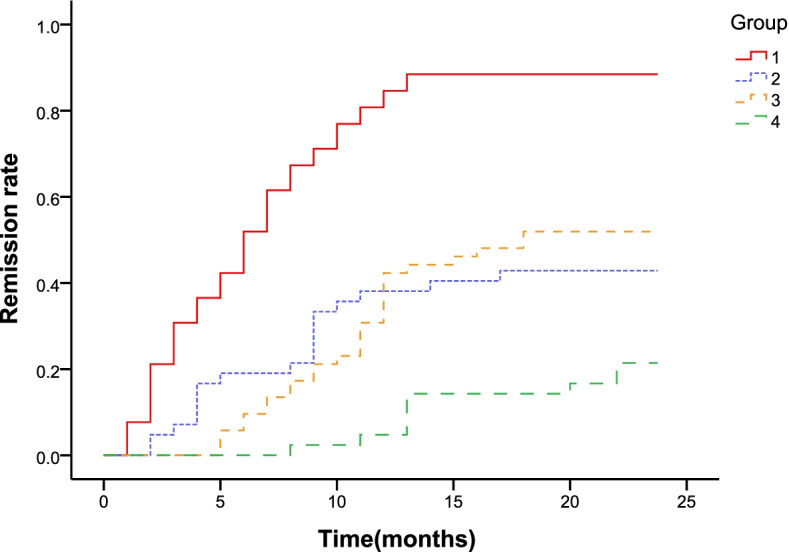
Remission rate is depicted for iMN patients in two groups at two years. The line 1 means total remission rate in low-level group, the line 2 means total remission rate in high-level group, the line 3 means complete remission rate in low-level group, the line 4 means complete remission rate in high-level group

All 94 patients received TAC as the initial immunosuppressive therapy. The follow-up period of patients ranged from 6–42 months (median, 19.5 months; mean, 19.76 ± 9.30 months). At the end of follow-up, 68.1% (64/94) patients achieved cumulative remission, 37 (39.4%) patients had CR and 27 (28.7%) patients had PR. For patients who achieved remission, the mean time to remission was 6.33 ± 3.94 months. The clinical remission rates of iMN patients in the two groups during the follow-up period are listed in Table 2. Six months after treatment with TAC, 51.9% (27/52) of the patients achieved remission (CR, 5 patients; PR, 22 patients) in the low-level group, and only 19% (8/42) of the patients achieved PR in the high-level group. The CR rate was significantly different between the groups during follow-up, but PR was not observed. Twelve months after treatment with TAC, 82.7% (43/52) of the patients in the low-level group achieved remission (including complete remission in 22 patients and partial remission in 21 patients), and the mean time was 6.52 ± 0.53 months. However, 12 months after treatment with TAC, only 38% (16/42) of the patients in the high-level group achieved remission (CR, 2 patients; PR, 14 patients), and the mean time was 9.86 ± 0.51 months. Figure 2 and Fig. 3 show TR and CR in two groups during follow-up based on the survival curve. Throughout the follow-up period, it was observed that the remission rate, either CR or PR, was lower in the high-level group. Two years after treatment with TAC, 88.5% (46/52) of the patients in the low-level group achieved remission (CR, 27 patients; PR, 19 patients), and the mean time to achieve CR was 16.94 ± 1.01 months. Meanwhile, 42.9% (18/42) of the patients in the high-level group achieved CR (mean, 22.07 ± 0.66 months). The remission rate of patients in the high-level group was lower than that in the low-level group, and it took longer to achieve remission. Therefore, TAC is not an optimal treatment option for iMN patients with serum anti-PLA2R antibody titers > 150 RU/ml.

**Table 2:** Clinical remission of iMN patients during the follow-up period

Time	Clinical remission	Low-level group(*n* = 52)	High-level group(*n* = 42)	*P* value
6 months	CR, n (%)	5 (9.6%)	0 (0%)	**0.047**
	PR, n (%)	22 (42.3%)	8 (19%)	0.140
	TR, n (%)	27 (51.9%)	8 (19%)	**0.001**
	NR, n (%)	25 (48.1%)	34 (81%)	** 0.001**
12 months	CR, n (%)	22 (42.3%)	2 (4.7%)	**< 0.001**
	PR, n (%)	21 (40.4%)	14 (33.3%)	0.313
	TR, n (%)	43 (82.7%)	16 (38%)	**< 0.001**
	NR, n (%)	9 (17.3%)	26 (62%)	**< 0.001**
18 months	CR, n (%)	27 (51.9%)	6 (14.3%)	**< 0.001**
	PR, n (%)	19 (36.6%)	12 (28.6%)	0.276
	TR, n (%)	46 (88.5%)	18 (42.9%)	**< 0.001**
	NR, n (%)	6 (11.5%)	24 (57.1%)	**< 0.001**
24 months	CR, n (%)	27 (51.9%)	9 (21.45%)	**0.002**
	PR, n (%)	19 (36.6%)	9 (21.45%)	0.085
	TR, n (%)	46 (88.5%)	18 (42.9%)	**< 0.001**
	NR, n (%)	6 (11.5%)	24 (57.1%)	**< 0.001**

Bold indicate *p* value < 0.05

Legend: *iMN* idiopathic membranous nephropathy, *low-level group* anti-PLA2R antibody titer ≤ 150 RU/ml, *high-level group* anti-PLA2R antibody titer > 150 RU/ml, *CR* complete remission, *PR* partial remission, *TR* total remission, *NR* no response

### Association between anti-PLA2R Ab levels and complication

The complications and drug-related side effects of iMN patients in two groups during the follow-up period are presented in Table 3. Twenty-seven infection-related events were accumulated, 11 cases in the low-level group, and 16 cases in the high-level group. The high-level group had more infection-related events, but the difference was not statistically significant (*P* = 0.065). There were nine cases of temporary hemodialysis due to severe edema (eight cases) or acute kidney injury (one cases) during follow-up (four cases in the low-level group and five cases in the high-level group). Among the 94 patients with iMN, 9 patients (9.6%) reached ESRD during follow-up (one patient in the low-level group and eight patients in the high-level group). The anti-PLA2R antibody titer in eight patients in the high-level group was greater than 288 RU/ml. Of those nine patients who reached ESRD, three died of ESRD during the follow-up period and had higher anti-PLA2R antibody titer (288RU/ml, 564RU/ml, and 883.42RU/ml, respectively). The high-level group showed a higher ratio of kidney dysfunction and death than the low-level group. Among the 94 iMN patients, 13 (13.8%) patients developed steroid diabetes during follow-up, six patients were in the low-level group, and seven patients were in the high-level group. There were no thromboembolic complications in either group.

**Table 3:** Complications and drug-related side effects of iMN patients in two groups during the follow-up period

Complications	Low-level group(*n* = 52)	High-level group(*n* = 42)	*P* value
Infection	11/55 (20%)	16/45 (35.6%)	0.065
acute kidney injury	1/52(1.9%)	1/42(2.4%)	0.697
ESKD	1/52 (1.9%)	8/42 (19%)	0.014
Died	0 (0%)	3/42 (7.1%)	0.086
Diabetes/glucose intolerance	6/52 (11.5%)	7/42(16.7%)	0.337
Thromboembolic complications	0 (0%)	0 (0%)	—

Legend: *iMN* idiopathic membranous nephropathy, *low-level group* anti-PLA2R antibody titer ≤ 150 RU/ml, *high-level group* anti-PLA2R antibody titer > 150 RU/ml, *ESRD* end-stage renal disease

## Discussion

This is the retrospective study to assess the efficacy and safety of TAC-based treatment in iMN patients with high titer anti-PLA2R levels (> 150RU/ml), who were recognized as high-risk patients with progressive loss of kidney function. We found that the remission rate, especially the CR rate, was much lower in the high-level group patients treated with TAC than in the low-level group. Moreover, the ESKD rate was significantly higher in the high-level group. The infection rate during the therapy period was higher in the high-level group; however, there was no statistical significance due to the limited number of cases in this study. Our results indicate that TAC-based treatment may not be an optimal choice for iMN patients with high anti-PLA2R levels.

As a calcineurin inhibitor, TAC stabilizes the podocyte cytoskeleton by interfering with calcium-dependent signaling pathways and inhibiting the activation and proliferation of T cells in vitro [25, 26]. Thus, TAC can reduce urinary protein excretion and is widely used in the treatment of nephrotic syndrome. A number of studies have shown that TAC, either monotherapy or in combination with low-dose corticosteroids, was effective in treating iMN patients [9, 27, 28]. Praga et al. used TAC monotherapy in iMN patients with normal kidney function and mean proteinuria over 12 months with a 6-month taper and compared them to conservation-treated patients. After 18 months, the total remission rate was 94% in the TAC group, but only 35% in the control group [9]. A retrospective study by Zhao Hu et al. recently showed that both TAC monotherapy and TAC combined with medium-dose prednisone in young patients aged between 15 and 40 years for 12 months led to 77.5%-80.4% remission rates [27].Thus, the KDIGO 2012 glomerular diseases guideline included TAC as one of the first-line immunosuppressive treatments for iMN patients [11], and KDIGO 2021 continued this proposition [10].

TAC has been widely used in iMN patients who require immunosuppressive therapy. The available clinical data are insufficient to guide physicians to assess who will have or not have a good response to the TAC-based treatment at diagnosis. Since Beck et al. reported the identification of PLA2R as a major antigenic target in iMN in 2009, a new era of this disease has been opened [12]. Now, anti-PLA2R antibody has been recognized as a biomarker that can establish the diagnosis of MN with high accuracy and without the associated risk of a renal biopsy [13–16]. Furthermore, anti-PLA2R antibody titers were closely associated with disease severity. For anti-PLA2R positive MN, a high level of anti-PLA2R antibody at diagnosis indicated an increased risk of progressive loss of kidney function [17–20], a low rate of spontaneous remission [17, 21–23], and vice versa. However, the relationship between the anti-PLA2R antibody titer and immunosuppressive treatment response has not been clearly demonstrated. Only a few clinical studies have compared the immunosuppressive treatment response between iMN patients with high and low anti-PLA2R antibody titers. Remuzzi et al. observed the treatment effect of rituximab in 81 anti-PLA2R positive MN patients, and found that the total remission rate in the lowest tertile group was 81.5%, which was approximately four times higher than that in the highest tertile group. Furthermore, the time to remission increased as the anti-PLA2R antibody titer increased [29]. Recently, Cattran et al. reported the data of the MENTOR study, which was conducted to compare the treatment response of rituximab and cyclosporine in iMN patients. In MENTOR, 52% of the patients treated with cyclosporine achieved CR or PR at 12 months. Subgroup analyses by anti-PLA2R tertile showed that only 18.8% of patients in the highest tertile group had CR or PR at 12 months, which was much lower than that in patients in the lower tertile in the MENTOR study when using cyclosporine [30]. Although TAC is another widely used CNI in the treatment of iMN, the treatment response in patients with a high anti-PLA2R antibody titer has not been reported. Our findings were consistent with the results of the MENTOR subgroup analyses: the total remission rate of TAC in the high-level group was only 19% (8/42) and 38% (16/42) at 6 months and 12 months, respectively, which was much lower than that in the low-level group. The mean remission time in the high-level group was 9.86 ± 0.51 months, which was longer than that of the low-level group.

Since the discovery of anti-PLA2R antibody as a pathogenic driver in nearly 70% of patients with iMN, a number of studies have confirmed the crucial role of anti-PLA2R levels in predicting the risk of disease progression [29, 31–37]. However, because the thresholds used to define a high titer varied in these studies, it remains unclear what specific antibody level should be used to predict the clinical outcomes, including the therapeutic response, length of immunosuppressive therapy, and the risk of progression. We defined anti-PLA2R antibody titer ≥ 150 RU/mL as high level according to the draft version of the 2020 KDIGO clinical practice guidelines on glomerular disease and up-to-date review of membranous nephropathy. We found that using 150 RU/mL as the cutoff value could predict the therapeutic response in iMN patients treated with TAC.

Adverse events related to TAC, such as abnormal glucose intolerance and nephrotoxicity, are important issues of concern [9]. In the present study, the rates of newly developed impaired glucose tolerance did not differ between the high- and low-level groups. However, the infection rate and death were higher in the high-level group, nevertheless, no statistical significance was found because of the limited sample size. There was a significant difference in the rate of progression to ESKD between the high-level and low-level groups, which was consistent with many previous studies [18–20]. No thromboembolic complications were reported in our study during the 24 months of follow-up period.

The present study had several limitations. As a single-center retrospective observational study, selection bias was inevitable, and the use of drugs among patients could not be controlled very strictly. The number in the two groups was different due to the real value of anti-PLA2R antibody titer detected by enzyme-linked immunosorbent assay (ELISA). Unfortunately, endpoint data were available from patient clinical records, which may have led to underestimation of adverse event rates. In addition, the sample size in our study was small.

In conclusion, our study proved that iMN patients with anti-PLA2R titers > 150 RU/mL at diagnosis showed unfavorable therapeutic response to TAC-based treatment. Moreover, TAC-based treatment may bring more benefits and is considered a better option for lower renal progression risk in iMN patients with anti-PLA2R titers ≤ 150 RU/ml. Further well-designed multicenter, large-scale randomized controlled trials are required to assess the efficacy and safety of TAC-based treatment in patients with high anti-PLA2R titers.

## Data Availability

The datasets generated and analyzed during the current study are available in the online link.[https://docs.google.com/spreadsheets/d/1SEnBhbKIlKQ34M0jmPcwyuzBmmZRGIDT/edit?usp=sharing&ouid=113189677743360538320&rtpof=true&sd=true].
