# Correction: Anti-phospholipase A2 receptor antibody levels at diagnosis predicts outcome of TAC-based treatment for idiopathic membranous nephropathy patients

**DOI:** 10.1186/s12882-022-02950-0

**Published:** 2022-09-26

**Authors:** Bihua Wang, Zhidan Zhu, Feng Huang, Haowen Huang, Luxia Tu, Ying Wang, Linfeng Zheng, Jing Zhou, Xin Wei

**Affiliations:** 1 Department of Nephrology, The First Affiliated Hospital of Nanchang University, 17 Yongwai Zhengjie, Nanchang City, 330006 Jiangxi China; 2 Nanchang University, Nanchang City, 330006 China; 3 Department of Nephrology, People’s Hospital of Ganjiang New District, Nanchang City, 330006 Jiangxi China; 4 Pathology Department, The First Affiliated Hospital of Nanchang University, Nanchang City, China

**Correction: BMC Nephrol 23, 306 (2022)**

**https://doi.org/10.1186/s12882-022-02914-4**

Following publication of the original article [1], the authors informed us that the first “Excluded” box in Fig. 1 is incomplete. The correct Fig. 1 is given below.

**Fig. 1:**
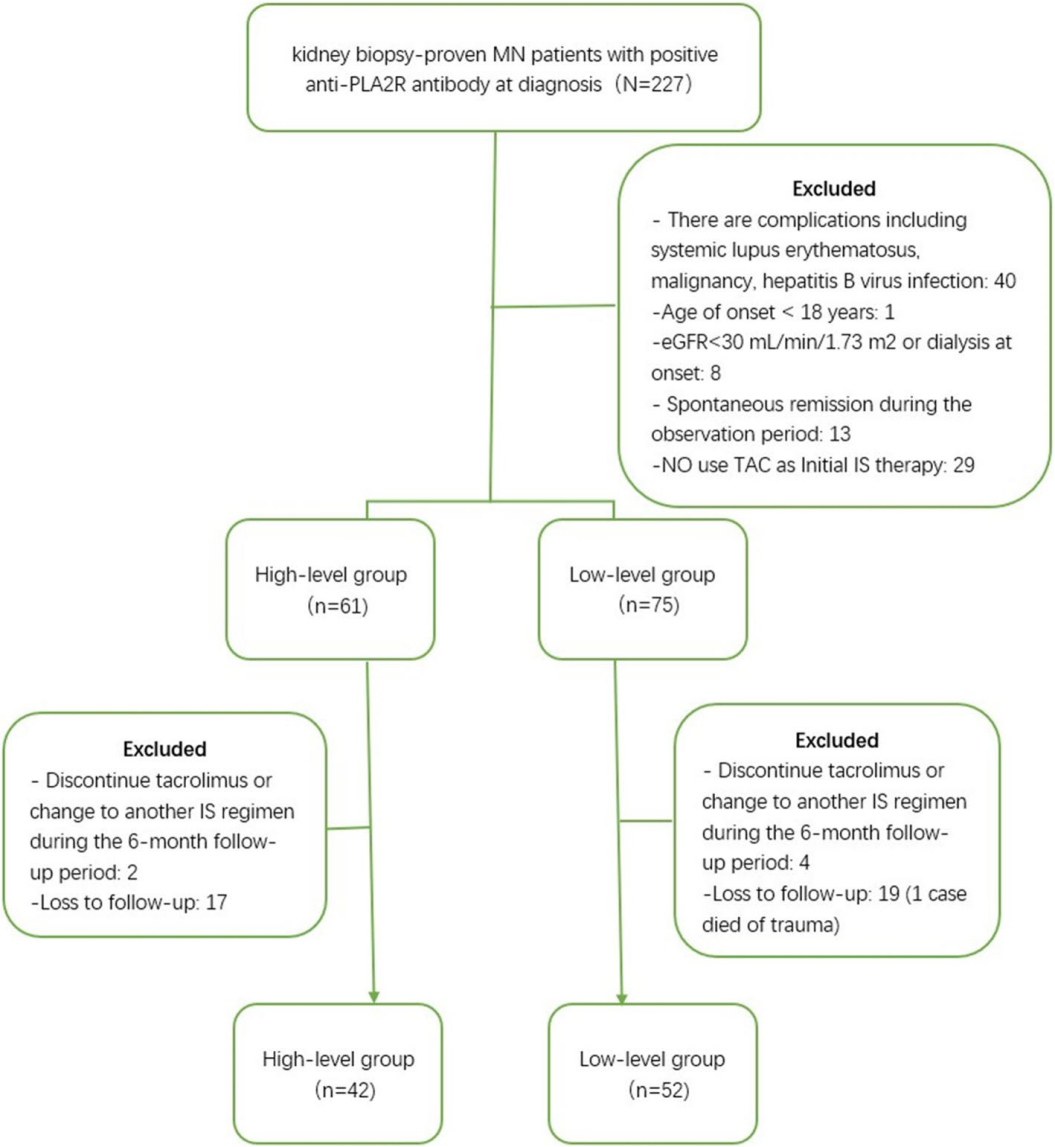
Inclusion flowchart of patients with idiopathic membranous nephropathy

The original article has been corrected.
